# Recombinant Production of Human Interleukin 6 in *Escherichia coli*

**DOI:** 10.1371/journal.pone.0054933

**Published:** 2013-01-23

**Authors:** Henrik Nausch, Jana Huckauf, Roswitha Koslowski, Udo Meyer, Inge Broer, Heike Mikschofsky

**Affiliations:** 1 Department of Agrobiotechnology, Agricultural and Environmental Faculty, University of Rostock, Rostock, Germany; 2 Bioserv GmbH, Rostock, Germany; Aligarh Muslim University, India

## Abstract

In this study, we compared basic expression approaches for the efficient expression of bioactive recombinant human interleukin-6 (IL6), as an example for a difficult-to-express protein. We tested these approaches in a laboratory scale in order to pioneer the commercial production of this protein in *Escherichia coli* (*E. coli*). Among the various strategies, which were tested under Research and Development (R&D) conditions, aggregation-prone IL6 was solubilized most effectively by co-expressing cytoplasmic chaperones. Expression of a Glutathion-*S*-Transferase (GST) fusion protein was not efficient to increase IL6 solubility. Alteration of the cultivation temperature significantly increased the solubility in both cases, whereas reduced concentrations of IPTG to induce expression of the T7lac-promotor only had a positive effect on chaperone-assisted expression. The biological activity was comparable to that of commercial IL6. Targeting the expressed protein to an oxidizing environment was not effective in the generation of soluble IL6. Taken together, the presence of chaperones and a lowered cultivation temperature seem effective to isolate large quantities of soluble IL6. This approach led to *in vivo* soluble, functional protein fractions and reduces purification and refolding requirements caused by downstream purification procedures. The final yield of soluble recombinant protein averaged approximately 2.6 mg IL6/liter of cell culture. These findings might be beneficial for the development of the large-scale production of IL6 under the conditions of current good manufacturing practice (cGMP).

## Introduction

Human interleukin-6 (IL6) is a cytokine with pleiotropic functions that is involved in a broad range of biological activities and was found to be associated with a vast number of severe inflammatory diseases, sepsis and rheumatoid arthritis. Pilot studies demonstrated that these diseases can be efficiently treated by targeting IL6, whereas standard antibiotic-based strategies fail [1]–[3]. Additionally, IL6 is a desirable clinical target for cancer therapy [4]. Consequently, functionally active recombinant expression of IL6 in large quantities is necessary, because extraction from human tissues is difficult and results in low protein yields [5].

Despite the availability and improvement of several alternative biopharmaceutical protein production platforms, *E. coli* offers advantages including growth on inexpensive carbon sources, rapid biomass accumulation, high cell-density fermentation and the ability to easily increase the production scale. Moreover, *E. coli* is well-characterized in terms of genetics and molecular biology [6].

IL6 has been expressed in *E. coli* by several research teams, but the resulting recombinant proteins tend to be aggregation-prone [7]–[9]. This might be a result of the fact that human IL6 naturally occurs not only as monomer but also forms multimeric aggregates of different molecular size [10]. May *et al*. [11] further demonstrated that these multimers possess a reduced biological activity, probably indicating that the intramolecular disulfide bridges were incorrectly formed and intermolecular crosslinking occurred. In *E. coli*, recombinant IL6 accumulated in inclusion bodies, but was biologically inactive, which might be a consequence of misfolded disulphide bridges as well [7]–[9]. For economic reasons, the renaturation protocol developed by 2 teams [5], [8] is not applicable to industrial large-scale production for medical applications. Furthermore, the resolubilization procedures may not fully restore the native protein fold and may reduce recombinant protein function [12].

In *E. coli*, solubilization of IL6 was achieved by fusion to maltose binding protein (MBP) and *N* utilization substance A (NusA) [4]. However, removal of the tag included several proteolytic cleavage and chromatography steps and was labor-intensive [13]. In addition, the overall yield of recombinant protein was dramatically reduced because only one-tenth of the overexpressed IL6 was recovered [13]. Therefore, this protocol does not meet industrial demands.

As an alternative strategy, Li *et al*. targeted IL6 to the periplasm by fusing the protein to α-hemolysin, achieved soluble and easily purified protein and demonstrated that the disulfide bonds formed correctly, generating a biologically active protein [14]. Unfortunately, the quantity of purified IL6 was very low, yielding 18 µg of IL6/liter of cell culture [14] compared to Lee *et al*. with 270 mg of IL6/liter of cell culture [13] or Spiridonova *et al*. with 3 mg of IL6/liter of cell culture [5]. Consequently, therapeutic application of IL6 is hampered by the lack of practical and economically feasible expression platforms that avoid laborious refolding steps and are compatible with industrial-scale protein production.

In this study, several common protein expression procedures were strategically combined in a laboratory screening assay, in order to define the basic parameter for the generation of large quantities of biologically active and soluble IL6 in *E. coli*. For the commercial production of protein therapeutics, the fermentation usually starts in a laboratory-scale bioreactor. Furthermore, the optimal growth and protein expression conditions are used to develop manufacturing scale in order to reach high productivity [15]. The *in vivo* synthesis of functional, soluble IL6 will significantly ease the downstream processing and thereby reduce the production costs under cGMP conditions. Generally, an oxidizing environment improves the expression of disulfide-containing proteins; therefore, IL6 was expressed in the *E. coli* strain Origami 2, harboring mutations in 2 major cytoplasmic oxidoreductases (trxB−/gor-) and promoting cytoplasmic disulfide bond formation. Alternatively, we investigated the effect of targeting IL6 to the periplasm of BL21 by fusing the secretion signal of the *E. coli* alkaline phosphatase (*phoA*). The concomitant expression of chaperones is another approach to produce soluble recombinant proteins in *E. coli*, because protein aggregation can result from chaperone limitation [16], [17]. In earlier studies, the aggregation of IL6 could be circumvented by fusion to MBP and NusA; nevertheless, this strategy requires the removal of the tag to restore biological activity to IL6 [4], [13]. To recapitulate the effect on solubility without the need to remove the tag, we fused IL6 to the Glutathion S-Transferase (GST) from *Schistosoma japanicum*, which does not interfere with the biological activity of the target protein [18]. Furthermore, adaptation of the cultivation conditions was combined with these strategies in order to maximize beneficial effects.

In addition to the *N*-terminal fusion tags (His-tag or GST-His-tag), which can be removed by proteolytic digestion, a *C*-terminal hexahistidine residue was fused to IL6 in all constructs for two reasons: (i) According to the manufacturer (Novagen pET system manual), the two His-tags improve the downstream purification efficiency in order to gain pure recombinant protein, free of endotoxin and other bacterial contaminations, required for GMP compliance. (ii) His-tags enable the site-specific PEGylation [19]. PEGylation is a clinically proven strategy to increase the circulation half-life of protein-based medicines [19]. Since the efficacy of protein-based agents can be compromised by their rapid clearance from the blood circulatory system [19], this might be particularly true for IL6. It is intended to use the recombinant protein for the production of IL6-specific antibodies. These antibodies shall be used to reduce the IL6 concentration in the blood of patients.

## Materials and Methods

### Construction of the Expression Plasmid and Overexpression Strains

**Figure 1:**
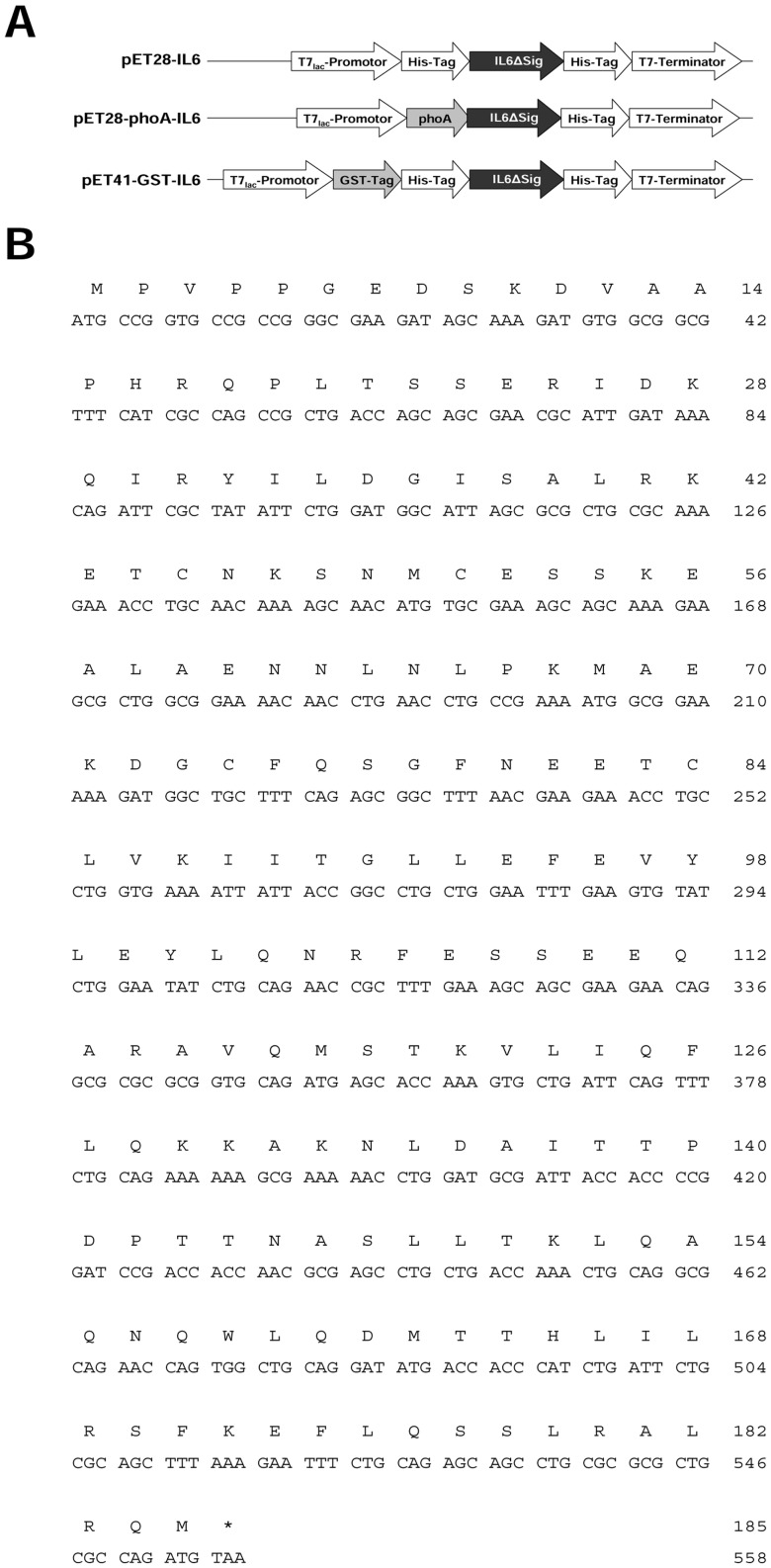
Constructs used to express the *IL6* gene. (A) Schematic representations of the of the vector; The vector backbones encode a kanamycin resistance gene and the pBR322 origin of replication. GST™-Tag: glutathione *S*-transferase (220 aa) from *Schistosoma japanicum*; His-Tag: 6 Histidine residues; T7-Tag®: initial 11 aa of the gene *10* protein from the T7-phage; phoA: periplasmic targeting signal of the *E. coli* alkaline phosphatase. Thrombin: 6 aa comprising a cleavage site for the thrombin protease. IL6ΔSig (184 aa) synthetic, codon-optimized gene sequences based on the mature protein sequence (without the ER-targeting signal); (B) Synthetic, codon-optimized coding region of the human cytokine IL6.

Based on the amino acid (aa) sequence of human interleukin 6 (Acc. No.: P05231-1) without the 28 N-terminal aa comprising the ER-targeting sequence, a synthetic coding sequence has been designed considering the optimal codon usage for *E. coli* (Fig. 1b).

The cassette was integrated in frame both into the vector pET28a (Novagen) using *Eco*RI/*Xho*I and into the vector pET41a using *Mun*I/*Xho*I digestion. The pET28a vector carries an N-terminal His-Tag/thrombin/T7-Tag in addition to a C-terminal His-Tag. The pET41a vector encodes an N-terminal 220 aa GST-fusion protein as well as a His-Tag/thrombin element and a second C-terminal His-Tag. To target the human protein to the periplasm, the upstream N-terminal coding portion of the pET28a vector was replaced with a codon-optimized synthetic sequence for the 21 aa signal peptide from the *E. coli* alkaline phosphatase (phoA; Acc. No.: AAA83893) using *Nco*I/*Eco*RI digestion.

All coding sequences were under the control of the inducible T7*lac* promotor and terminator. The different constructs were depicted in Figure 1a.

The helper plasmids pBB540 and pBB5542, encoding the cytoplasmic chaperones DnaK/DnaJ/GrpE and GroEL/GroES, were provided by Dr. Bernd Bukau [20]. The vector pTUM4.1, expressing the periplasmic chaperones DsbA, DsbC, SurA and FkpA, was provided by Dr. Arne Skerra [21].

The combination of different expression vectors and *E. coli* strains used in this work are listed in Table 1.

**Table 1:** Recombinant strains used in this work.

Host strain	Recombinant plasmids	Fusion Tag
Origami 2	pET28-IL6ΔSig	n- & c-terminal His Tag
		T7-Tag
BL21	pET28-IL6ΔSig	n- & c-terminal His Tag
		T7-Tag
BL21	pET28-phoA-IL6ΔSig	c-terminal His-Tag
		
BL21	pET28-IL6ΔSig	n- & c-terminal His Tag
	pBB540/pBB542	T7-Tag
BL21	pET28-phoA-IL6ΔSig	c-terminal His-Tag
	pTUM4.1	
BL21	pET41-GST-IL6ΔSig	n-terminal GST-Tag
		n- & c-terminal His-Tag

Competent BL21 (DE3) and Origami 2 (DE3) cells (Novagen) were transformed by electroporation with the different pET-vectors for expression of the target genes. These recombinant strains were subsequently transformed using the helper plasmids pBB540/pBB542 or pTUM4.1. Cells were grown in presence of appropriate antibiotics to ensure the maintenance of all plasmids.

### Protein Expression

Single colonies from the transformed cells were used to inoculate a pre-culture of 5 ml of LB medium supplemented with the appropriate antibiotics. Liquid cultures were grown at 37°C and 220 rpm overnight. From this pre-culture, 1 ml was used to inoculate 100 ml of LB medium containing antibiotic. The main cultures were incubated at 37°C and 220 rpm until they reached an OD600 of 0.5. For protein expression at 37°C, 1 mM IPTG was immediately added, and cells were incubated at 37°C for 4 h. For protein expression at 22°C, the cultivation temperature was changed from 37°C to 22°C, and after a 30 min incubation, 1 mM IPTG was added. Cells were further grown at 22°C overnight. Overnight cell cultures were pelleted, the medium was removed and the cell pellet was resuspended in the same volume of fresh medium supplemented with 200 µg/ml chloramphenicol. After a 2-h incubation of the culture at 22°C, the cultures were centrifuged and the pellet was stored at −28°C until further processing.

### Western Blot Analysis

The frozen bacterial pellet was resuspended in 5 ml of lysis buffer (50 mM NaH_2_PO_4_, 300 mM NaCl, 10 mM imidazole, pH 8.0), and 1 mg/ml lysozyme was added. The suspension was incubated on ice for 2 h with gentle shaking, followed by sonication in water for 5 min. Representative, 50 µl of the cell debris was separated from the supernatant by centrifugation. SDS sample buffer was added to both fractions, which were subsequently boiled and loaded on a 15% SDS PAGE. For Western blot analysis, proteins were transferred to polyvinylidene fluoride (PVDF) membranes for 2 h at 1 mA/cm^2^. Subsequent steps were performed at room temperature. Membranes were blocked with 5% non-fat dry milk in phosphate-buffered saline containing 0.1% Tween-20 (PBST) overnight. Membranes were incubated with a 1∶1,000 dilution in PBST of a mouse monoclonal anti-IL6 antibody (Dianova, Hamburg/Germany) for 2 h. Following a triple washing step with PBST, membranes were incubated with a 1∶10,000 dilution of a peroxidase-conjugated goat monoclonal anti-mouse-IgG antibody (Dianova, Hamburg/Germany) for 2 h. After 5 PBS washes, the probed proteins were visualized with Biomax Light film (Kodak, Sigma-Aldrich, Taufkirchen/Germany) with lab-made chemiluminescent substrate (ECL Solution I: 100 mM Tris (pH 8.5), 2.5 mM Luminol, 400 µM p-Coumaric acid; ECL Solution II: 100 mM Tris (pH 8.5), 5.4 mM H_2_O_2_; ECL solutions were mixed immediately before use).

### Enzyme-linked Immunosorbent Assay (ELISA)

Cell lysate, supernatant and cell debris fractions for the Western blot assay were also used for Bradford and ELISA. The protein concentration was measured by the Bradford method (1976) using the Pierce reagent and bovine serum albumin (BSA) as standard (Thermo-Scientific, Bonn/Germany). The quantification of recombinant IL6 was performed with a commercial IL6-ELISA-Kit (Human IL-6 Ready-SET-Go ELISA (eBioscience; Cat. 88-7066-86)) according to the manufacturer’s instructions. In brief, 96-well plates were coated with a mouse anti human IL6-specific antibody at a final concentration of 1 µg/ml at RT overnight. Following 5 washes with PBS with 0.05% Tween-20, the plates were incubated with 100 µl of leaf extract at an appropriate dilution at RT for 2 h. After another washing step, plates were incubated with the corresponding biotinylated detection antibody at RT for 1 h. Subsequent to washing, hybridization with streptavidin conjugated to horseradish peroxidase at RT for 30 min was performed. Finally, the plate was incubated with the substrate tetramethylbenzidine (TMB) at RT for 15 min in the dark. The reaction was terminated with 250 mM sulfuric acid. Extinction was measured at 450 nm in a Synergy HT multi-detection reader (Bio-Tek, Bad Friedrichshall/Germany).

### Determination of the Biological Activity of Recombinant IL6

The biological activity was evaluated with the hybridoma proliferation assay using an IL6-dependent murine hybridoma cell line (B-9) and according to the protocol “Bioassay for IL6 using the B9 cell line“ from the method manual “Cytokine Cell Biology, a Practical Approach” by Fran Balkwill (third edition 2000). After 3 to 4 days of cell line cultivation with the addition of recombinant IL6, tetrazolium salt MTT (3-(4,5-Dimethylthiazol-2-yl)-2,5-diphenyltetrazolium-bromid) was added. Depending on the cell density, MTT was converted to formazan by the mitochondrial enzyme succinate dehydrogenase. The photometric extinction at 550 nm correlates to the cell proliferation and therefore to the concentration of biologically active IL6.

The concentration of recombinant protein inducing half-maximal growth stimulation was defined as the EC_50_ value. The specific activity was calculated by dividing the measured photometric extinction through the concentration of IL6 (in pg/ml) that was applied.

## Results

### Comparison of *E. coli* (DE3) Strains BL21 and Origami 2 for Recombinant Protein Expression

**Figure 2:**
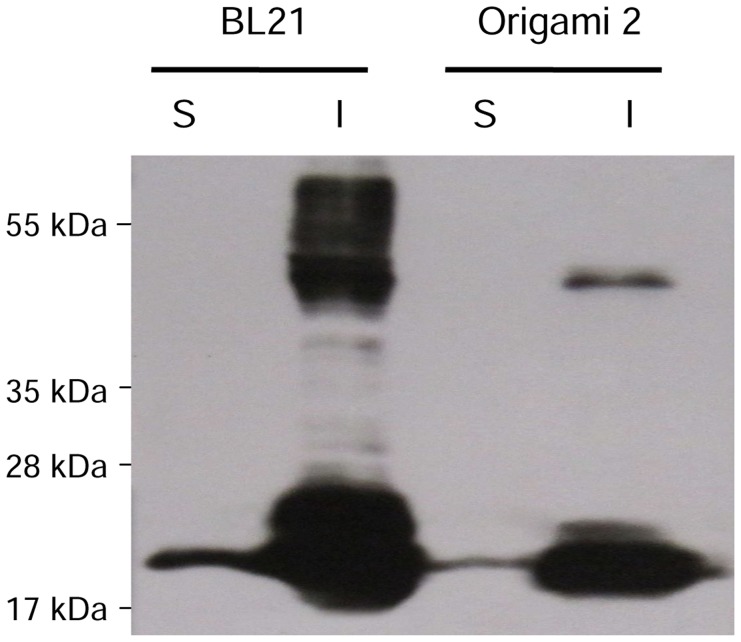
Western blotting analysis of the IL6-expressing *E. coli* strains BL21 and Origami 2. (S) soluble and (I) insoluble fraction of IL6.

The pET28-IL6ΔSig plasmid was introduced into the *E. coli* strains BL21 (lon^−/^ompT^-^) and Origami 2 (trxB^−/^gor^-^), which are characterized by either a reducing (BL21) or an oxidizing cytoplasm (Origami 2). To determine the effect of oxidizing cytoplasm on the organization of the disulfide bonds in IL6, the expression patterns from each strain were compared by Western blot. When expression was induced with 1 mM IPTG at 37°C for 4 h, most of the recombinant proteins were deposited in the insoluble fraction of the cell lysate both as mono- and multimeres (Fig. 2). Higher amounts of soluble IL6 were produced in BL21 compared to Origami 2 (Fig. 2– lane S). In addition, the total amount of IL6 was significantly higher in the BL21 strain, because both mono- and multimeric IL6 in the inclusion body fraction was reduced in Origami 2 (Fig. 2– lane I). Moreover, we observed that BL21 had a significantly higher growth rate. The Origami 2 strain needed two to three times longer in order to reach the OD_600 nm_ of 0.5 for the induction of the recombinant protein expression (data not shown). Because Origami 2 did not improve production of soluble protein or the biological activity (as described below), we choose *E. coli* BL21 as the expression strain due to its vigorous growth pattern.

### Expression in the Cytoplasm and Periplasmic Space of *E. coli* BL21

To combine the growth kinetics of *E. coli* BL21 with an oxidizing environment for native folding of the intramolecular disulfide bonds, IL6 protein was translocated to the periplasm. The translocation was accomplished by substituting the N-terminal His- and T7-Tag in the corresponding pET28-IL6ΔSig vectors with the signal peptide encoded by the endogenous alkaline phosphatase from *E. coli*.

**Figure 3:**
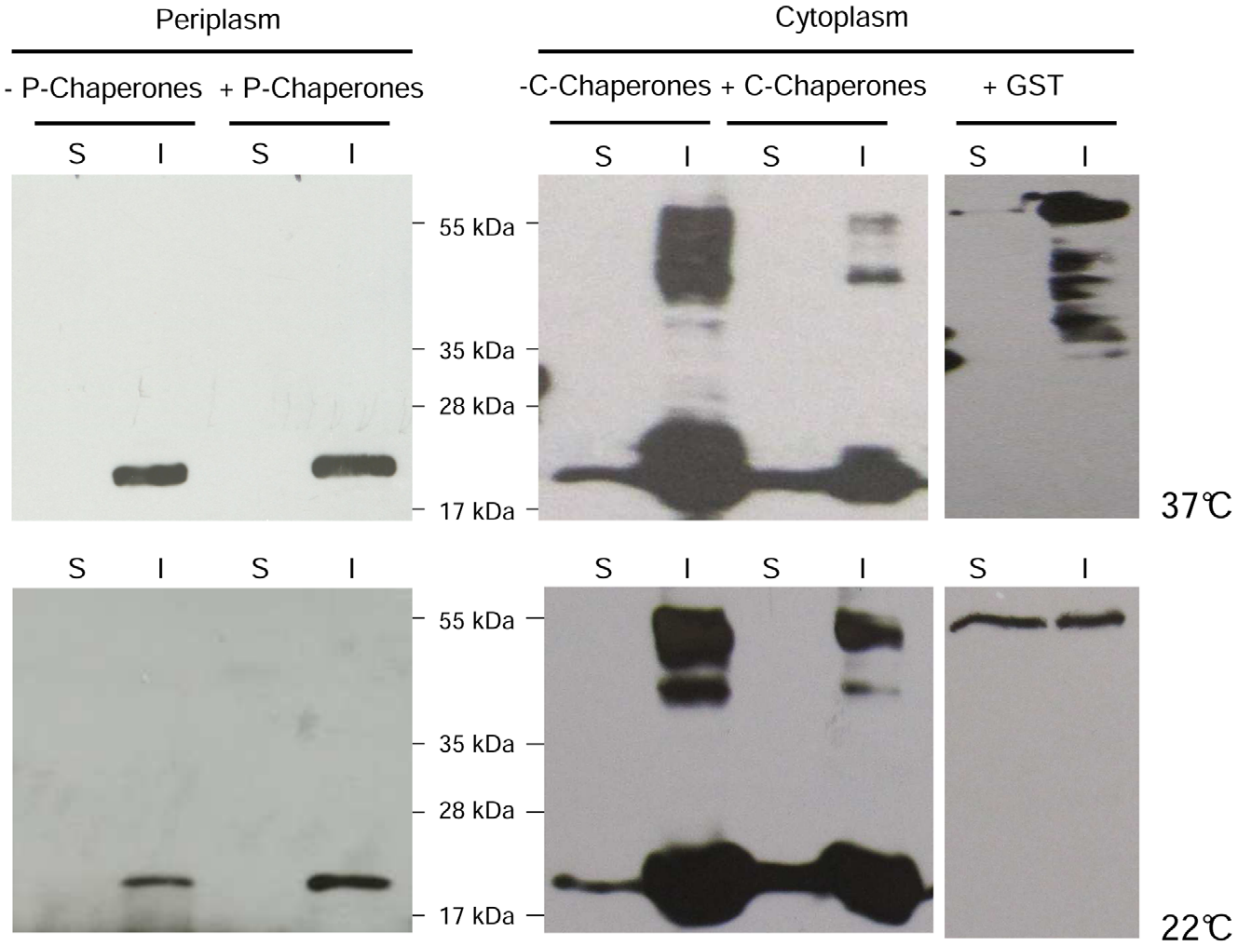
Western blotting analysis of the IL6 expression in the cytoplasm and periplasm of *E. coli* BL21. (S) soluble and (I) insoluble fraction of IL6, expressed with and without the concomitant overexpression of endogenous cytoplasmic chaperones DnaK, DnaJ, GrpE, GroES, GroEL (C-Chaperones) or periplasmic chaperones DsbA, DsbC, SurA, FkpA (P-Chaperones) at 37°C and 22°C, respectively. Additionally, IL6 was expressed fused to GST in the cytoplasm.

As shown in Figure 3, this strategy was unsuccessful. Soluble IL6 was not localized to the periplasm. In addition, the amount of IL6 accumulated in inclusion bodies was significantly reduced. However, it must be noted that the formation of high molecular weight aggregates of IL6 was impaired by translocation to the periplasmic compartment.

### Concomitant Expression of Cytoplasmic and Periplasmic Chaperones

The effect of concomitant overexpression of a set of cytoplasmic chaperones (DnaK, DnaJ, GrpE, GroES, GroEL) as well as key periplasmic folding factors (DsbA, DsbC, SurA, FkpA) was investigated. The overexpression of both sets of chaperones differentially affected the folding pattern of IL6. The periplasmic chaperones had no detectable influence (Fig. 3; Periplasm+P-Chaperones) on both soluble and insoluble recombinant proteins. However, there was a reproducible enhancement of soluble IL6 when the cytoplasmic chaperones were overexpressed. In addition, both insoluble IL6 monomers and multimeric aggregates were reduced by this approach, decreasing the overall amount of IL6 deposited in inclusion bodies.

### Expression of an IL6-GST Fusion Protein

To fuse GST to IL6, the plasmid pET41-GST-IL6ΔSig was constructed. As shown in Figure 3 (Cytoplasm+GST), the tag was not suitable to track IL6 in the soluble fraction. Similar quantities of IL6 were accumulated into inclusion bodies when compared to the chaperone overexpression strain.

### Effect of Cultivation Temperature and Inducer Concentration

A well-known approach to limit the *in vivo* aggregation of recombinant proteins is cultivation at a reduced growth temperature [16]. The expression of periplasm-targeted IL6, both with and without the additional chaperone expression, was not affected by reduction of the cultivation temperature down to 22°C (Fig. 3; Periplasm). However, a lowered temperature increased the levels of soluble IL6 in the presence of overexpressed cytoplasmic chaperones (Fig. 3; Cytoplasm+C-Chaperones) when compared to the strain without folding factors. Though, the amount of aggregated IL6 was not reduced and the multimerization was not affected in both strains. A beneficial effect was also observed for the GST fusion protein. There were fewer inclusion bodies, and soluble GST-IL6 was enriched when compared to expression at 37°C (Fig. 3; Cytoplasm+GST). Nevertheless, the total amount of soluble GST-IL6 was significantly lower compared to the soluble IL6 achieved with improved folding due to chaperone expression.

Normal physiological protein expression rates can be achieved for heterologous expressed proteins with a reduced inducer concentration [16]. Because the expression of recombinant proteins is fully induced at concentrations above 0.4 mM IPTG, expression has been induced with 0.1 mM compared to the conventionally used 1 mM IPTG as proposed by de Marco [20].

**Figure 4:**
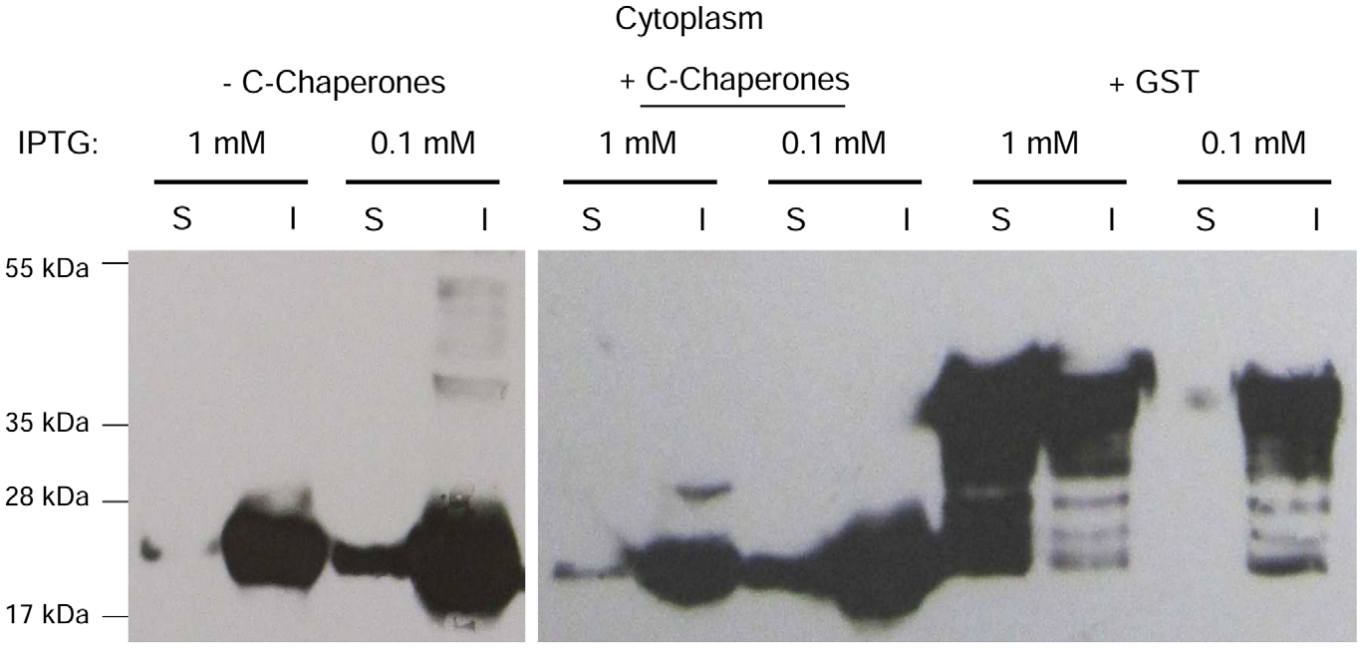
Western blotting analysis of the IL6 expression with reduced IPTG concentrations. (S) soluble and (I) insoluble fraction of IL6, expressed with either 1 mM or 0.1 mM IPTG at 22°C in the cytoplasm of the *E. coli* BL21 strain with and without the concomitant overexpression of endogenous cytoplasmic chaperones or in fusion to GST.

The strains in which IL6 is localized in the cytoplasm showed improved solubilization when the IPTG concentration was reduced (Fig. 4). The level of inclusion bodies was increased in strains with and without engineered chaperones, whereas the formation of multimeric aggregates at approximately 32 kDa was reduced. For the GST-fusion protein, the effect was reversed. Under these conditions, no soluble recombinant protein was detected (Fig. 4;+GST 0.1 mM) and the insoluble fraction was unaffected.

### Quantification and Evaluation of the Biological Activity of *E. coli*-derived Human IL6

To determine the expression levels of soluble recombinant IL6, the fraction of total soluble proteins (TSP) was extracted from cell culture pellets by lysozyme digestion and centrifugation. The different fractions of the cell lysate were tested by both ELISA and the bioassay. Biological activity is defined as the concentration of recombinant protein that induces a half-maximal response (EC_50_ value). Because the GST-IL6 fusion protein displayed a limited solubility, this protein was not tested in the bioassay.

The ELISA data were in agreement with the results of the Western blots. The yield of recombinant IL6 was highest in the transgenic *E. coli* BL21 strain with co-expressed chaperones, presumably due to a high transgen expression level and enforced chaperone activity in the cytoplasm. As shown in Table 2, the average yield was 2.6 mg of IL6/liter of cell culture, which corresponds to 0.5% of the TSP. In one of 5 experiments, the yield reached up to 0.73% of TSP. However, this strain produced the most potent recombinant IL6 with an EC_50_ value of 28 pg/ml (Tab. 2). Compared to the reference standard, recombinant IL6 from this strain displayed equal biological activity. The strain without concomitant overexpression of chaperones led with 0.4% of TSP to a slightly reduced yield compared to the strain with chaperone assistance; however, the biological activity was reduced, with an EC_50_ value 150% higher at 41 pg/ml. In line with that was the specific activity of recombinant IL6 derived from this strain lower (6.3*10^−3^) compared to the strains with the co-expressed chaperones (13.5*10^−3^; table 2). We observed a biological activity only for insoluble IL6 when produced at 22°C, but not at 37°C (data not shown). Nevertheless, the activity was lower compared to the soluble IL6 and we were not able to determine the EC_50_-value. The EC_50_ values of the soluble and insoluble fraction of the transgenic Origami 2 strain were similar to the BL21 strain without the co-expressed chaperones. The soluble and insoluble fraction of the *E. coli* strains with the control vector pET28a did not induce any cell proliferation in the bioassay.

**Table 2:** Quantification of soluble IL6 by ELISA and evaluation of biological activity.

BL21 strain	Portion of TSP	Biological activity	Specific activity of 1 pg IL6
	[%]	EC_50_ value of IL6 [pg/ml]	
IL6-Standard	–	25.0 (±2.7)	14.84*10^−3^ (±1.7)
pET28-IL6ΔSig	0.44 (±0.2)	41.03 (±3.1)	6.30*10^−3^ (±2.8)
pET28-IL6ΔSig	0.54 (±0.3)	27.76 (±2.2)	13.45*10^−3^ (±1.7)
+ Chaperones			

## Discussion

The use of recombinant proteins for most biotechnological and biomedical applications requires soluble protein fractions, because biological activity is often conformation-dependent [22]. However, many expressed eukaryotic proteins accumulate in inclusion bodies due to a lack of post-translational modifications, which play a crucial role in protein folding, stability and biological activity [23]. Resolubilization and refolding procedures are laborious in many instances and frequently lead to low yields [16].

These protein expression caveats hold true for the expression of human interleukin 6 (IL6). When expressed in *E. coli*, IL6 is prone to aggregation [7]–[9] and is non-functional. Resolubilization protocols developed for IL6 [5], [8] are not economically feasible for industrial large-scale production. Similarly, MBP-IL6 and NusA-IL6 fusion proteins are highly soluble *in vivo* [4], but require an intricate separation from the tag necessary to restore biological activity [13]. Expression of IL6 in the periplasm generated biologically active IL6 molecules, but the expression level was quite low [14].

Therefore, there remains a need to develop an effective and cost-efficient method to express biologically active IL6, in order to provide a large-scale production system of IL6 under cGMP condition. To modify IL6 expression in *E. coli*, preventing protein aggregation in inclusion bodies in order to generate soluble and properly folded protein, we investigated a broad range of strategies, summarized in Table 3. Since these are fundamental expression strategies, the findings will be relevant for large-scale commercial fermentation.

**Table 3:** Evaluation of expression parameters on the solubilization of recombinant IL6 yield (-: no soluble target protein; +: <10%; ++: 10–25%; +++25–50%; ++++: >50% of total recombinant protein localized to the soluble fraction) and biological activity (n.i: not experimentally investigated.

Expression strategy	description	Temperature/Inductor	Yield of soluble IL6	Bioactivity of soluble IL6	Specific activity of 1 pg IL6
				(EC_50_ value)	
1	Origami 2 - cytoplasm	37°C	+	∼ 40 pg/ml	7.22*10^−3^ (±3.1)
		22°C	n.i.	n.i	
2	BL21 - periplasm	37°C	–	–	
		22°C	–	–	
3	BL21– periplasm+P-Chaperones	37°C	–	–	
		22°C	–	–	
4	BL21 - cytoplasm	37°C	+	∼ 40 pg/ml	6.48*10^−3^ (±3.2)
		22°C	++	n.i.	
		22°C - 0.1 mM IPTG	++	41.03 pg/ml (±3.1)	6.30*10^−3^ (±2.8)
5	BL21– cytoplasm+C-Chaperones	37°C	++	n.i.	
		22°C	++	n.i.	
		22°C - 0.1 mM IPTG	+++	27.76 pg/ml (±2.2)	13.45*10^−3^ (±1.7)
6	BL21– cytoplasm+GST	37°C	+	n.i.	
		22°C	++	n.i.	
		22°C - 0.1 mM IPTG	+	n.i.	

Although the *E. coli* Origami strain can be an advantageous host for eukaryotic proteins, such as the tissue plasminogen activator [24], the serine protease inhibitor HF6478 [25], the lutropin/choriogonadotropin receptor [26] or the antigen binding fragments [27], our experiments led to a clear preference for the *E. coli* strain BL21 to express IL6. First, in our experiments BL21 demonstrates a growth pattern compared to Origami 2 that is more suitable for commercial high cell density fermentation. This is in agreement with previous reports that recorded that the growth of K12 derivates, such as Origami 2, are negatively effected by the high acetate accumulation during cultivation and that B strains are more efficient in the utilization of glucose [28]–[33]. Second, BL21 is deficient in 2 major cytoplasmic proteases, which promotes the accumulation of a high level of recombinant protein [34], [35]. Both factors promote high yield of the target protein. In fact, in our study total amount soluble and insoluble IL6 was higher in the BL21. Additionally, in a comparative study of various *E. coli* strains, BL21 exhibited the lowest stress response to high synthesis rates of foreign protein [36]. Third, we did not observe any improvement of the solubility and biological activity of IL6 when produced in the Origami 2 strain. Fourth, B strains are highly desirable in industry [15]. However, IL6 is already partially soluble when expressed using conventional conditions (Tab. 3–4), but most of the recombinant protein is loaded into inclusion bodies. Interestingly, high-molecular-weight bands occur in the insoluble fraction, as observed for human IL6 *in vivo* [10]. This is consistent with that expressed human IL6 in *E. coli* [8]. No improvement was observed when IL6 was expressed in an oxidizing environment (Tab. 3–2). Surprisingly, the translocation led to complete accumulation in inclusion bodies and an overall reduction in yield. This is contrary to Li *et al*. [14], in which targeting of IL6 to the periplasm by fusion to α-hemolysin led to significant solubilization. However, the fusion protein was not removed after translation and may have increased the solubility. In addition, tagged IL6 was not detectable in the corresponding ELISA under non-denaturating conditions but was detectable in Western Blots under denaturating conditions [14]. This discrepancy suggests that the soluble chimeric protein is not functional. Furthermore, the overall yield of periplasmic IL6 was significantly higher than cytoplasmic IL6. Low yields of periplasmic IL6 may be caused by the limited capacity of the secretion pathway, which is known to limit the expression of recombinant proteins [37], [38].

According to previous reports, engineering of periplasmic chaperones can solubilize aggregates of recombinant proteins such as the human plasma retinol-binding protein [21] or the human granulocyte colony-stimulating factor [39]. This strategy was pursued in our study (Tab. 3–3) but was not effective. Xu *et al*. were unable to identify recombinant PalB in the periplasm and contributed this effect to the inherent instability of the recombinant protein [40]. This may also be true for IL6, because IL6 is rapidly metabolized by serum peptidases after release into the blood stream [10]. IL6 is stabilized *in vivo* by binding to a soluble receptor in the blood [41].

In contrast, the concomitant overexpression of cytoplasmic chaperones, which has also been demonstrated to be a powerful tool for the solubilization of eukaryotic proteins [6], [42], led to an increased level of soluble IL6 (Tab. 3–5). Lowering the cultivation temperature and reducing the inducer concentration potentiated this effect. Amazingly, insoluble IL6 deposited in inclusion bodies was increased and showed a biological activity under these conditions, but not when produced at 37°C. This might indicate that the ‘22°C’ inclusion bodies contain a higher percentage of correctly folded but aggregated protein. The correctly folded IL6 is probably more resistant to proteolytic degradation, which might explain the increased amount of insoluble IL6. Nevertheless, the activity was inferior compared to soluble IL6. This is in agreement with May *et al*. [11], who demonstrated that aggregated human IL6 is less active compared to the monomeric variant. However, our results are consistent with previous reports in which reduced cultivation temperatures were beneficial for the expression of a broad range of proteins [20]. The research group of de Marco *et al*., for instance, tested 64 proteins and improved the solubility of 70% of them using this strategy [20]. It is hypothesized that the deposition of recombinant proteins in inclusion bodies is a stress response caused by overtaxed folding machinery [16]. Using a viral promoter, which leads to expression levels well above the physiological norm, the quantity of newly expressed recombinant protein exceeds the number of available chaperones involved in the folding process [16]. The hydrophobic stretches of newly synthesized proteins are highly amenable to aggregation [43]. Consequently, increasing the chaperone concentration is a suitable remedy. According to de Marco, the reduction of cultivation temperature decelerates overall metabolism, and therefore the quantity of newly synthesized protein, leading to a further equalization of chaperone capacity and recombinant protein concentration. Additionally, it has been shown that hydrophobic interactions and therefore aggregation is impeded at lower temperatures [44].

The bioassay further demonstrated that not only was the yield of soluble IL6 improved by additional chaperones, but the biological activity was improved as well. IL6 derived from the strain with overexpressed chaperones exhibited a lower EC_50_ value (28 pg/ml) compared to the strain without assistance (41 pg/ml). The final yield of soluble, active IL6 averaged approximately 2.6 mg/liter of cell culture or 0.5% of the TSP. The inclusion bodies possessed no biological activity, in concordance with previous reports [7]–[9].

Another method to influence the folding pattern of the target protein is by fusing them to proteins that are themselves highly soluble [45]–[47]. In this study we employed GST, because it has solubilization and protein-stabilizing features and is known not to interfere with the biological activity of IL6 [18]. However, the impact of GST on the solubility of IL6 was limited (Tab. 3–6). This may be because GST (26 kDa) is similar in size to IL6 (21 kDa). In previous reports, proteins that are significantly larger than the target, such as NusA (54 kDa) or MBP (40 kDa), have the strongest effect on solubility of the passenger protein [48]–[51]. Kim *et al*. [4] confirmed this assumption for IL6. In this study, IL6 was fused to MBP, NusA, Trx and Ubiquitin (Ubi; 8.5 kDa). Solubilization of IL6 was possible only when using larger proteins, such as MBP and NusA.

Furthermore, solubilization of the fusion protein GST-IL6 was enhanced by lowering cultivation temperatures. Hydrophobic interactions are less pronounced at lower temperatures, possibly inhibiting undesired aggregation [44].

This is the first report of functional human IL6 expressed in *E. coli* without the requirement of a fusion tag or refolding process. Nevertheless, a 10 fold higher yield could be achieved by fusion with MBP [13]. Since the removal of MBP required an expensive separation and refolding process to restore biological activity of soluble IL6, analysis regarding which procedure is more cost-efficient for the large-scale production of soluble IL6 must be performed.

Chaperone engineering in combination with an overall reduction of the protein biosynthesis rate was optimal for the heterologous expression of IL6 in *E. coli* (Tab. 3). The final yield of soluble IL6 increased to 0.54% of total cellular protein, which corresponds to 2.6 mg of IL6/liter of cell culture. Fortification of the folding machinery improved both the yield and the activity of IL6 (Tab. 2).

### Conclusions

In this study, we demonstrated that the adaptation of the protein synthesis rate and the protein folding capacity of the chaperone network were crucial for the production of difficult-to-express proteins in *E. coli*, such as human IL6. This can be accomplished by the modification of various parameters. Both the reduction of the cultivation temperatures and the concentration of the inductor for the promoter used for the expression of the target reduce the synthesis rate of the recombinant protein. On the other hand, the concomitant expression of chaperones improves the folding capacity of the host. Combining these strategies leads to a remarkable synergistic effect. This might represent an alternative to the *in vivo* solubilization through the use of fusion tags, which are avoided in industry since the fusion protein is not the protein of interest, or an alternative to *in vitro* recovery and refolding of IL6 from inclusion bodies. However, it needs to be investigated if soluble IL6 can be efficiently purified using the His-tag. Nevertheless our findings are relevant for commercial large-scale fermentation, since high downstream processing costs are major constraint for the commercial application of microbial expression platforms [23].
